# Carbapenem-resistant *Pseudomonas aeruginosa* T3SS virulence genes and correlation between virulence and drug resistance and molecular epidemiology studies

**DOI:** 10.3389/fphar.2025.1591724

**Published:** 2025-06-18

**Authors:** Jianhua Tang, Jiekun Pu, Linbo Zhao, Xuli Zhan, Qianqian Pang, Xiangyan Meng, Rui Feng, Haojun Fan

**Affiliations:** ^1^ School of Disaster and Emergency Medicine, Tianjin University, Tianjin, China; ^2^ The First Affiliated Hospital of Hebei North University, Zhangjiakou, Hebei, China; ^3^ The Fourth Hospital of Hebei Medical University and Hebei Tumor Hospital, Shijiazhuang, Hebei, China

**Keywords:** carbapenem-resistant, *Pseudomonas aeruginosa*, T3SS, virulence genes, tolerance, molecular epidemiology carbapenem-resistant, molecular epidemiology

## Abstract

**Objective:**

This study aimed to investigate the relationship between the carriage and expression of virulence genes associated with the type III secretion system (T3SS) in carbapenem-resistant *Pseudomonas aeruginosa* (CRPA) and their impact on virulence, drug resistance, and molecular epidemiological patterns in clinical isolates.

**Methods:**

CRPA strains isolated from the First Hospital Affiliated to Hebei North College between April 2022 and February 2023 were analyzed. Antimicrobial susceptibility was tested using the microbroth dilution method. Second-generation high-throughput sequencing was employed to detect four virulence genes (exoU, exoY, exoS, and exoT) and β-lactamase genes in CRPA strains with T3SS. Multilocus Sequence Typing (MLST) was performed to determine the sequence types (ST) of the strains. The *Galleria mellonella* larvae assay was used to evaluate strain virulence and qPCR was used to measure the expression levels of virulence genes.

**Results:**

Resistance to imipenem was observed in 100% of the strains, whereas resistance to polymyxin B was the lowest at 6.7%. The highest detection rate of T3SS virulence genes was for exoT (100%), followed by exoS, exoY (88.9%), and exoU (11.1%). Twenty-two ST types were identified, with ST 244 being the most prevalent (31.1%). In the *G. mellonella* assay, five strains exhibited high virulence, 25 strains exhibited medium virulence, and 15 strains showed low virulence. Statistically significant differences in resistance rates to piperacillin/tazobactam, levofloxacin, and amikacin were observed among the CRPA strains with varying virulence levels (P < 0.05).

**Conclusion:**

T3SS virulence genes and virulence levels in CRPA strains were correlated with antibiotic resistance. CRPA strains producing blaOXA-101 and blaPER-1 spread throughout the region.

## 1 Introduction

*Pseudomonas aeruginosa* (*P. aeruginosa*) is a prevalent opportunistic pathogen with diverse metabolic pathways and minimal environmental requirements, enabling its survival under hostile conditions (Rossi et al., 2021). It primarily colonizes the respiratory tract and cutaneous mucosa and is associated with a broad spectrum of respiratory diseases in humans, making it a leading cause of hospital-associated infections (Constantino-Teles et al., 2022). According to 2023 data from the Bacterial Resistance Surveillance Network, which includes 201,740 isolates from 73 hospitals, *P. aeruginosa* ranks fifth, comprising 7.5% of the total isolates. The resistance rates of *P. aeruginosa* to imipenem and meropenem have increased notably in recent years. Carbapenem antibiotics, typically the first-line treatment for *P. aeruginosa* infections and the last defense against severe cases, have been overused, contributing to the rise in carbapenem-resistant *P. aeruginosa* (CRPA). The growing prevalence of CRPA has made selecting effective antimicrobial therapies more challenging (Tenover et al., 2022), highlighting the need to optimize or develop new treatment strategies for CRPA infections.

The pathogenic mechanisms of CRPA are highly complex, encompassing intrinsic, acquired, and adaptive antibiotic resistance mechanisms as well as a broad genomic array of virulence factors (Sultan et al., 2021). Major resistance mechanisms in CRPA include the production of antibiotic-inactivating enzymes, overexpression of efflux pumps, and modification or loss of outer membrane porins (Meletis et al., 2012; Pang et al., 2019). Notably, the production of antibiotic-inactivating enzymes is particularly significant, as CRPA can acquire these genes through mobile genetic elements, facilitating their rapid spread (Meletis et al., 2012; Ohadian et al., 2020). Moreover, the production of these inactivating enzymes varies by region (Reyes et al., 2023). CRPA virulence is closely tied to its encoded secretion systems, particularly type I to VI secretion systems (Glanville et al., 2021). Among these, type III secretion system (T3SS) is the most complex and potent virulence factor. Through T3SS, bacteria acquire essential nutrients for survival and reproduction while secreting virulence proteins into the surrounding environment or directly targeting eukaryotic cells (Horna and Ruiz, 2021). T3SS primarily secretes four effector proteins: ExoT, ExoS, ExoY, and ExoU. ExoT exhibits bifunctional virulence properties, inducing apoptosis in various forms (Wood et al., 2015) and impairing normal phagocytic functions in host cells (Vourc’h et al., 2017). ExoS, highly homologous to ExoT, contains two active structural domains that contribute to apoptosis by inhibiting cell division, causing acute tissue damage, and facilitating bacterial dissemination (Foulkes et al., 2022). ExoY increases the intracellular concentration of cNMP, disrupts the host endothelial barrier, and triggers both acute and chronic infections (Silistre et al., 2021). ExoU induces rapid host cell lysis and death by targeting the plasma membrane and is often linked to multidrug resistance and poor patient prognosis (Foulkes et al., 2019). Multilocus sequence typing (MLST) is a widely used technique for bacterial molecular typing, which allows for the identification of sequence types (ST) of strains. Investigating the epidemiological patterns of CRPA is crucial for preventing the spread of high-risk clones and strengthening infection control measures (Zhang et al., 2022).

Integrating the study of virulence genes with antibiotic resistance and molecular epidemiology provides valuable insights into the adaptability and transmission dynamics of pathogens in various environments. This holistic approach not only aids in tracking the spread of high-risk clonotypes but also informs the development of more effective treatment strategies and infection control measures. It plays a vital role in curbing the proliferation of antimicrobial-resistant pathogens and in improving public health outcomes. Furthermore, this methodology supports the rapid identification of outbreak sources, accelerates public health responses, and enhances infectious disease management. Few studies have explored the correlation among CRPA T3SS virulence genes, strain virulence, and antibiotic resistance. This study aimed to analyze the relationship between the carriage and expression of CRPA T3SS virulence genes, strain virulence, antibiotic resistance, and molecular epidemiological patterns of CRPA in our hospital. These findings are expected to provide valuable insights into addressing antibiotic resistance and optimizing infection control strategies for CRPA.

## 2 Materials and methods

### 2.1 Source of strains

A total of 45 unique CRPA strains were collected from clinical isolates at the First Affiliated Hospital of Hebei North University between April 2022 and February 2023, excluding duplicates from the same patient. CRPA was identified using an automated microbiological bacterial identification/pharmacological sensitivity analysis system and mass spectrometry. Quality control strains, including *P. aeruginosa* ATCC 27853 and *Escherichia coli* ATCC 25922, were maintained in our laboratory. *P. aeruginosa* standard strain PAO1 was obtained from the Beijing Beina Chuanglian Biotechnology Research Institute.

### 2.2 Instruments and reagents

Columbia blood agar was sourced from Beijing Solebao Technology Co., Ltd., and MacConkey agar from Shanghai Zeye Biotechnology Co., Ltd. *Galleria mellonella* (*G. mellonella*) larvae were purchased from Tianjin Huiyuide Biological Co., Ltd. The microsyringes used in the experiments were obtained from Shanghai Gao Dove Microsyringe, and drug sensitivity reagents were obtained from Shanghai Yuanye Biotechnology Co., Ltd. Additionally, 96-well plates and sterile Petri dishes were acquired from Shanghai Muchen Biotechnology Co. Ltd. The fully automatic bacterial identification/pharmacological sensitivity analysis system was supplied by BioMérieux (France). A biological safety cabinet was obtained from Jinan Xinbeisi Biotechnology Co., Ltd., and RNA extraction, reverse transcription, and qPCR kits were obtained from Xavier Biotechnology Co., Ltd.

### 2.3 Whole-genome sequencing

#### 2.3.1 Annotation of resistance and virulence genes

DNA was extracted and purified from the strains for whole-genome sequencing (WGS). DNA libraries were prepared according to the manufacturer’s protocol using the Illumina NEBNext Ultra II FS DNA Library Preparation Kit (New England Biolabs, Ipswich, MA, USA). The library samples were sequenced using the MiSeq platform with a 300bp pair-end WGS protocol. The quality of raw reads was assessed using FastQC v0.11.5 (de Sena Brandine and Smith, 2019) and reads were trimmed using Fastp v0.20.0 (de Sena Brandine and Smith, 2019). The final assembly quality was evaluated using QUAST v5.0.2 (Gurevich et al., 2013), and annotation was performed using Prokka v1.14.6, with default settings. The assembled genome sequences were uploaded to the RAST website (https://rast.nmpdr.org/rast.cgi) and the Resfinder database (https://cge.cbs.dtu.dk/services/Resfinder/) for genome annotation and resistance gene comparison. Virulence gene data were analyzed using the Virulence Factor Database (VFDB) (Liu et al., 2019), with queries conducted via ABRicate v1.0.0 (https://github.com/seemann/ARBicate).

#### 2.3.2 MLST typing analysis

The bacterial whole-genome sequence was submitted to the PubMLST website (https://pubmlst.org/organisms/pseudomonas-aeruginosa/) to obtain the corresponding allele numbers for the seven housekeeping genes of *P. aeruginosa* (acsA, aroE, guaA, mutL, nuoD, ppsA, and trpE). The seven allele numbers were concatenated in the order acsA, aroE, guaA, mutL, nuoD, ppsA, and trpE to form the allele profile. This profile was uploaded to the website for comparison, resulting in the ST type of each bacterial isolate. In cases where an allele lacked a corresponding number or ST type, the information was uploaded to the website for review, and a new number was assigned.

### 2.4 Strain identification and drug susceptibility testing

The experimental strains were selected for inoculation and cultivation using the three-zone delineation method on agar medium for 18–20 h. Fresh colonies were used to assess the antimicrobial susceptibility of CRPA to 13 commonly used clinical drugs using the microbroth dilution method. The interpretation of drug sensitivity results and statistical analysis followed the 2023 Clinical and Laboratory Standards Institute (CLSI) guidelines.

### 2.5 *Galleria mellonella* virulence test

The *G. mellonella* larvae infection model was established based on established protocols (Admella and Torrents, 2023). This experiment involved 45 CRPA strains and *G. mellonella* larvae aged 5–6 weeks, measuring 2–3 cm in body length, weighing 200–300 mg, with milky-white skin, responsiveness to mechanical stimuli, and rapid recovery after inversion. For each strain, 10 larvae were used, with a 10 µL injection of a bacterial suspension (1 × 10^6^ colony-forming units [CFU]) into the left hind foot of the larvae, administered using a 25 µL microsyringe. Successful inoculation was confirmed if no liquid leaked from the larvae within 5–10 s of injection.

**FIGURE 1:**
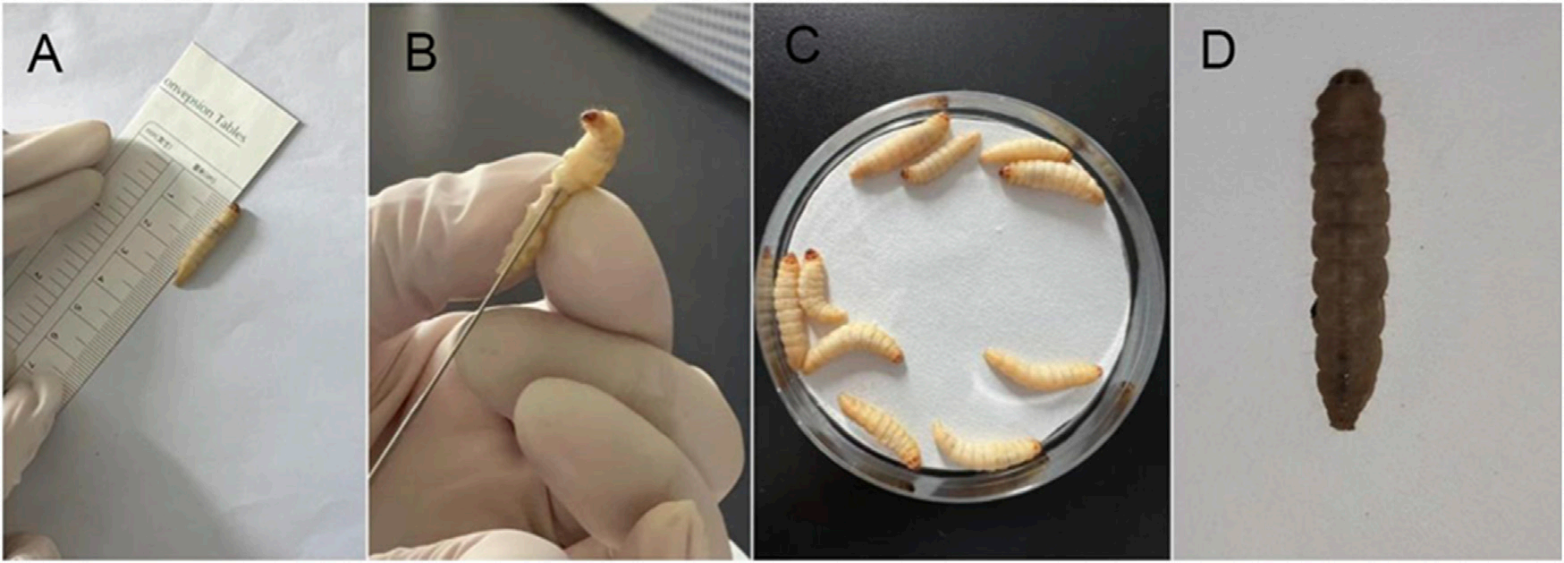
*Galleria mellonella* larvae virulence test. **(A)** Experimental animal standard: Larvae aged 5–6 weeks, body length 2–3 cm, body weight 200–300 mg, milk-white color, sensitivity to mechanical stimulation, and ability to quickly flip over when inverted. **(B)** Inoculation method: A 25 μL microinjector was used to inject 1 × 10^6 CFU of bacterial suspension into the larva via the left hind leg. Observe the larva for 5–10 s; if no liquid leaks from the larva’s surface, the inoculation is successful. **(C)** Observation conditions: Incubation in a dark environment at 37°C. Observations were recorded every 2 h. **(D)** Death determination: if the larva does not respond to repeated physical stimuli, it is determined to be dead.

The group injected with the bacterial suspension represented the experimental group, while the control group consisted of larvae injected with sterile PBS buffer or no treatment. The injected larvae were incubated in the dark at 37°C, and observations were recorded at 2-h intervals. Survival curves were generated by averaging the results of three replicates. Larvae were considered deceased if they failed to respond to repeated physical stimuli (Figure 1). *P. aeruginosa* standard strain PAO1, known for its medium virulence (Grace et al., 2022), served as the control strain. The log-rank (Mantel-Cox) test was used to compare the survival rates of *G. mellonella* larvae infected with other bacterial strains and PAO1. Strains with no significant survival rate differences from PAO1 (P > 0.05) were classified as medium virulence. Strains showing significant differences in survival rate (P < 0.05) and survival curves above PAO1 were designated as having low virulence, whereas strains with survival curves below PAO1 were categorized as highly virulent.

### 2.6 Detection of virulent gene expression levels

The experimental strains were cultured overnight in liquid medium on a constant temperature shaker. RNA was extracted using a kit and its concentration and purity were assessed. The lowest RNA concentration required for the system formulation was determined, with purity values ranging from 1.9 to 2.1, which met the necessary criteria. Reverse transcription was performed followed by qRT-PCR. Primers for qRT-PCR were designed in this study, with the internal reference gene 16S rRNA based on the literature (Bustos et al., 2022), and synthesized by Sangong Bioengineering (Shanghai) Co. The primer sequences are shown in Table 1. The PAO1 standard strain was used as the control. The relative expression levels of virulence genes in each strain were calculated using the relative quantification method (2^−ΔΔCT^ method), following fluorescence quantification.

**TABLE 1:** Sequence of virulence factor primers for qRT-PCR.

Gene	Primer sequence 5′→3′
*exoY*	F: ATG​ACC​GCC​GAT​TAT​GAC​CTC​TTC
	R: CCA​TAG​AAT​CCG​TCC​TCG​CTC​AG
*exoT*	F: CGC​CCT​TTA​CCT​CGC​TCT​CTA​C
	R: CCG​CCA​GCC​AGC​ATG​TAC​TC
*exoS*	F: GAG​AGC​GAG​GTC​AGC​AGA​GTA​TC
	R: ACT​TCG​GCG​TCA​CTG​TGG​ATG
*exoU*	F:GGAGCGAGTCGGTGAACATCTG
	R:GTTGAGCAACACTGGTGAGCATAC
16S rRNA	F:TCTAAGGAGACTGCCGGTGA
	R:CAGCTGCGATCCGGACTAC

Note: Primer specificity for exoY, exoT, exoS, and exoU was confirmed through BLAST, analysis against the *Pseudomonas aeruginosa* genome (NCBI, database) to ensure the absence of cross-reactivity. The 16S rRNA, primers were sourced from reference Bustos et al. (2022), where their specificity had been previously validated.

### 2.7 Statistical analysis

Statistical analysis was performed using the SPSS software (version 26.0). Chi-square (χ^2^) or Fisher’s exact tests were used for categorical data analysis. The survival rate of *G. mellonella* larvae was calculated and graphically presented using the GraphPad Prism 8. The log-rank (Mantel-Cox) test was used to compare the survival rates between *G. mellonella* larvae infected with different bacterial strains. Statistical significance was set at p < 0.05.

## 3 Results

### 3.1 Types of strain specimen

**FIGURE 2:**
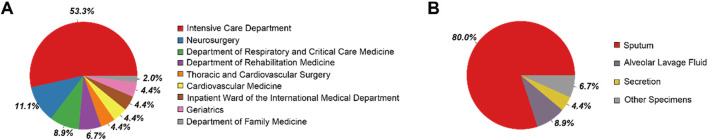
**(A)**. Distribution of strains by department; **(B)**. Types of strain specimen. **(A)** Departmental distribution of 45 CRPA clinical isolates, with the majority sourced from the intensive care department (53.3%, 24/45), and additional isolates originating from various other departments. **(B)** Classification of specimen types, including sputum (80.0%, 36/45), alveolar lavage (8.9%, 4/45), secretions (4.4%, 2/45), and other specimen types (6.7%, 3/45).

The clinical isolates of the 45 CRPA strains were predominantly collected from the intensive care unit, accounting for 53.3% (24/45) of the samples, with additional strains sourced from various other departments (Figure 2A). The specimen types were categorized as sputum (80.0%, 36/45), alveolar lavage (8.9%, 4/45), secretions (4.4%, 2/45), and other (6.7%, 3/45), as shown in Figure 2B.

### 3.2 Genomic Features and virulence gene analysis

**FIGURE 3:**
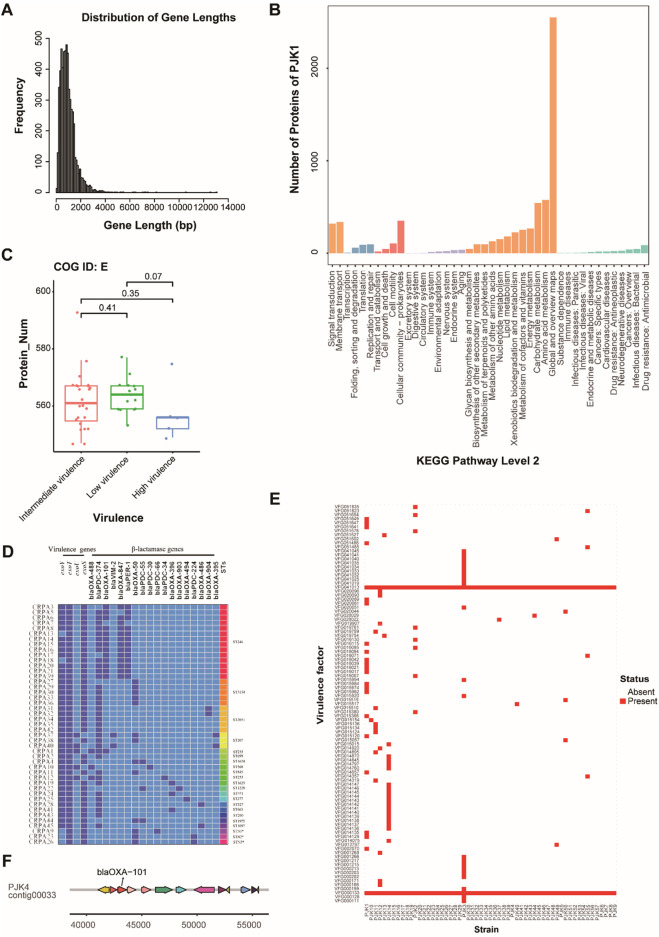
Genomic Features and Functional Analysis of CRPA Isolates. **(A)** Distribution of gene length across all CRPA isolates, showing the frequency of genes at different length intervals (bp). Most of the genes fell within the 500–2,000 bp range. **(B)** KEGG pathway analysis showing the functional distribution of annotated genes in strain PJK1, categorized by major biological processes and molecular functions. **(C)** Box plot showing the distribution of genes involved in amino acid transport and metabolism (COG ID: E) across the strains with different virulence levels. A significant decrease in gene number was observed in the high-virulence strains. **(D)** Heatmap of specimens, virulence genes, and STs of the 45 isolates. Dark blue represents the presence of the gene, light blue represents its absence, and * represents a newly discovered ST types. **(E)** Heatmap showing the presence (red) and absence (white) of virulence factors across all CRPA strains. Two universally present virulence factors (VFG000133 and VFG041013) were highlighted. **(F)** Example of genomic context showing the location of blaOXA-101 and the associated genomic region in strain PJK4.

The whole genome sequencing of 45 CRPA isolates revealed considerable genomic diversity. Gene prediction identified 5,433 and 6,935 protein-coding genes per isolate, respectively, with a median of 6,069 genes. The gene length distribution followed a typical bacterial pattern, with most genes ranging between 500–2000 bp (Figure 3A). Functional annotation showed extensive coverage across the Cluster Orthologous Group (COG), Gene Ontology (GO), and Kyoto Encyclopedia of Genes and Genomes (KEGG) databases (Figure 3B). Notably, the analysis of COG functional categories indicated that genes involved in amino acid transport and metabolism (COG category E) were negatively correlated with virulence levels, with high-virulence strains containing fewer genes in this category (P < 0.05) (Figure 3C).

Second-generation high-throughput sequencing revealed varying distributions of the T3SS virulence genes among the isolates (Figure 3D). The exoT virulence factor had the highest carriage rate at 100% (45/45), followed by exoS and exoY at 88.9% (40/45), whereas exoU exhibited the lowest rate at 11.1% (5/45). Three distinct virulence genotype combinations were identified: exoY^+^/exoS^+^/exoT^+^/exoU^−^ (77.8%, 35/45), exoY^+^/exoS^−^/exoT^+^/exoU^+^ (11.1%, 5/45), and exoY^−^/exoS^+^/exoT^+^/exoU^−^ (11.1%, 5/45). Beyond the T3SS genes, genome-wide analysis of virulence factors revealed 11–397 virulence-associated genes per isolate. Two virulence factors, VFG000133 and VFG041013, were conserved across all strains, suggesting their critical roles in CRPA pathogenicity (Figure 3E). Genomic context analysis revealed pathogenicity islands near blaOXA-101 in specific strains (PJK4, 7, 9, 16, 17, 21, 23, 24, and 42), indicating potential co-evolution of virulence and resistance traits (Figure 3F).

Eighteen β-lactamase genes were detected, with blaPDC-374 being the most prevalent (77.8%, 35/45), followed by blaOXA-101 (31.1%, 14/45), blaOXA-847 (31.1%, 13/45), blaPER-1 (31.1%, 13/45), and blaOXA-50 (31.1%, 13/45). MLST analysis identified 22 ST subtypes, with ST244, ST3134, and ST2651 being the most prevalent, at 31.1% (14/45), 11.1% (5/45), and 11.1% (5/45), respectively. The high-risk clonotypes ST244, ST235, and ST277 were also detected in this study (Figure 3D).

### 3.3 Comprehensive analysis of antimicrobial resistance

Drug sensitivity testing of 45 clinical CRPA isolates revealed diverse resistance profiles. CRPA exhibited the highest susceptibility to polymyxin B (93.3%), followed by amikacin (64.4%), and ceftazidime/avibactam (53.3%). The resistance rates to other antimicrobials exceeded 60%, with imipenem showing complete resistance (100%) (Table 2). These patterns demonstrate significant strain-to-strain variation, indicating the presence of diverse resistance mechanisms.

**TABLE 2:** Statistics on CRPA resistance (%).

Drug category	Drug resistance situation		
	Intermediate	Sensitive	Resistance
Piperacillin/tazobactam	48.9 (22/45)	20.0 (9/45)	31.1 (14/45)
Ceftazidime	55.6 (25/45)	4.4 (2/45)	40.0 (18/45)
Cefepime	55.6 (25/45)	6.7 (3/45)	37.8 (17/45)
Aztreonam	68.9 (31/45)	15.6 (7/45)	17.8 (8/45)
Imipenem	100.0 (45/45)	0	0
Meropenem	77.8 (35/45)	17.8 (8/45)	4.4 (2/45)
Ciprofloxacin	46.7 (21/45)	15.6 (7/45)	37.8 (17/45)
Levofloxacin	62.2 (28/45)	24.4 (11/45)	17.8 (8/45)
Amikacin	33.3 (15/45)	2.2 (1/45)	64.4 (29/45)
Ceftazidime/avibactam	46.7 (21/45)	—	53.3 (24/45)
Imipenem/relebactam	33.3 (15/45)	26.7 (12/45)	40.0 (18/45)
Cefttolozane/tazobactam	33.3 (15/45)	8.9 (4/45)	57.8 (26/45)
Polymyxin B	6.7 (3/45)	93.3 (42/45)	—

**FIGURE 4:**
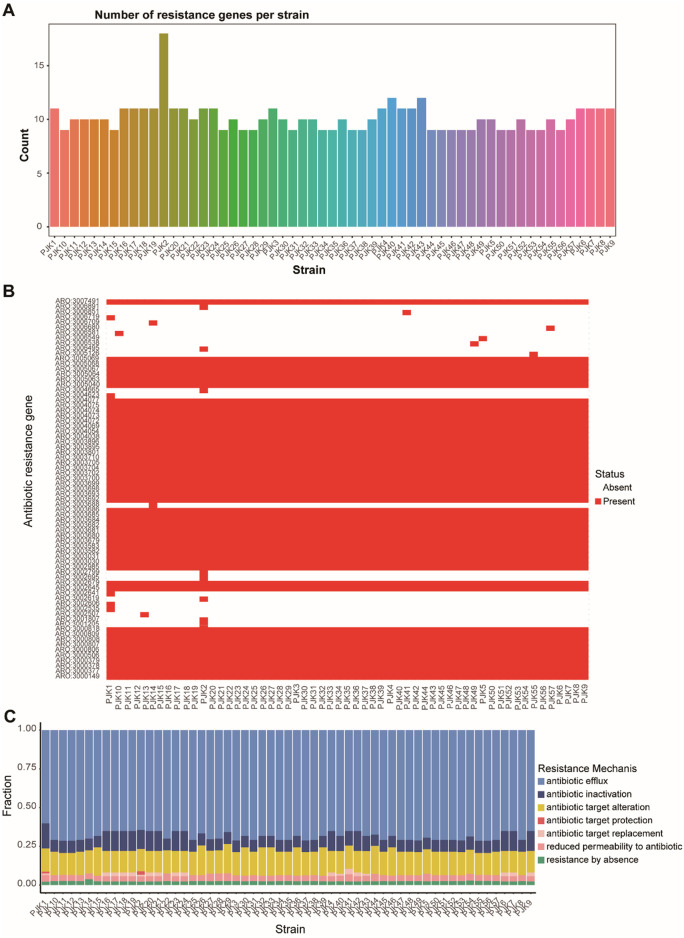
Comprehensive Analysis of Antimicrobial Resistance Patterns. **(A)** Bar plot showing the distribution of resistance genes across all CRPA strains, revealing variations from 8 to 15 resistance genes per strain. **(B)** Heatmap displaying the presence (dark) and absence (light) of specific antibiotic resistance genes across all strains, demonstrating the diversity of the resistance determinants. **(C)** Stacked bar chart showing the distribution of different resistance mechanisms across strains, including antibiotic efflux, inactivation, target alteration, protection, replacement, reduced permeability, and absence of resistance.

Genome-wide analysis identified 116 distinct resistance genes across all isolates, with individual strains carrying 8–18 resistance genes. PJK2 contains the highest number of resistance genes (18), suggesting that it is a particularly resistant strain (Figure 4A). Resistance mechanism analysis revealed multiple concurrent strategies, including prophylactic “antibiotic target protection” (present in PJK1 and PJK2) and “antibiotic target replacement” (identified in 18 strains including PJK1, PJK2, PJK4, PJK6, PJK7, PJK9, PJK16-21, PJK23, PJK24, and PJK40-43) (Figure 4B). The distribution of resistance genes was strongly correlated with the phenotypic resistance patterns. Strains harboring both antibiotic target protection and target replacement mechanisms exhibited significantly higher minimum inhibitory concentrations (MICs) for multiple antibiotics (P < 0.05). The analysis also identified common and unique resistance genes across the isolates, revealing core resistance determinants shared by all strains as well as strain-specific mechanisms (Figure 4C).

Resistance patterns among the three virulence genotypes (exoY^+^/exoS^+^/exoT^+^/exoU^-^, exoY^+^/exoS-/exoT^+^/exoU^+^, and exoY^-^/exoS^+^/exoT^+^/exoU^-^) were analyzed and showed significant differences. The exoY^+^/exoS^+^/exoT^+^/exoU^-^ group exhibited significantly higher resistance rates to aztreonam, meropenem, and levofloxacin than the other groups (P < 0.05) (Table 3). This suggested a potential association between specific virulence genotypes and resistance profiles.

**TABLE 3:** Resistance rate (%) of CRPA to commonly used antimicrobial drugs in different virulotypes.

Drug category	Genotype			*P* Value
	*exoY* ^*+*^ */exoS* ^*-*^ */exoT* ^*+*^ */exoU* ^*+*^	*exoY* ^*-*^ */exoS* ^*+*^ */exoT* ^*+*^ */exoU* ^*-*^	*exoY* ^*+*^ */exoS* ^*+*^ */exoT* ^*+*^ */exoU* ^*-*^	
Piperacillin/tazobactam	20.0 (1/5)	80.0 (4/5)	48.6 (17/35)	0.184
Ceftazidime	80.0 (4/5)	80.0 (4/5)	48.6 (17/35)	0.249
Cefepime	80.0 (4/5)	80.0 (4/5)	48.6 (17/35)	0.249
Aztreonam	40.0 (2/5)	20.0 (1/5)	80.0 (28/35)	**0.008**
Imipenem	100.0 (5/5)	100.0 (5/5)	100.0 (5/5)	—
Meropenem	60.0 (3/5)	40.0 (2/5)	85.7 (30/35)	**0.034**
Ciprofloxacin	20.0 (1/5)	60.0 (3/5)	45.7 (16/35)	0.451
Levofloxacin	20.0 (1/5)	40.0 (2/5)	71.4 (25/35)	**0.046**
Amikacin	20.0 (1/5)	20.0 (1/5)	37.1 (13/35)	0.301
Ceftazidime/avibactam	60.0 (3/5)	40.0 (2/5)	45.7 (16/35)	0.880
Polymyxin B	0 (0/5)	0 (0/5)	8.6 (3/35)	1.000

The bolded numbers in the tables represent P values that are less than or equal to 0.05.

### 3.4 Results of the *Galleria mellonella* virulence test

**FIGURE 5:**
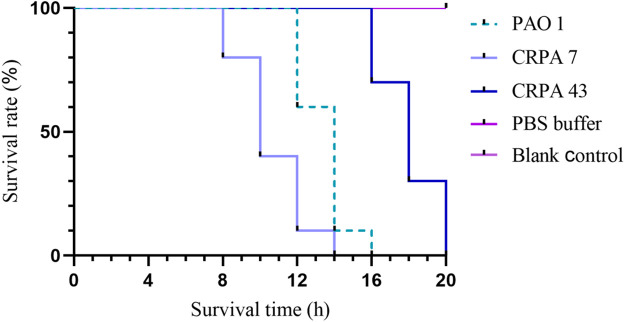
*Galleria mellonella* larvae survival curves. *Pseudomonas aeruginosa* standard strain PAO1 served as a control strain with a medium level of virulence. Representative strains CRPA7 (high virulence) and CRPA43 (low virulence) were selected to generate survival curves for *Galleria mellonella*.

The 45 clinical CRPA isolates were classified into three virulence categories (low, medium, and high) based on the *G. mellonella* virulence test, as outlined in Section 2.5 of the Materials and Methods. The results revealed that 33.3% (15/45), 55.6% (25/45), and 11.1% (5/45) of the strains exhibited low, medium, and high virulence, respectively. CRPA7, representing a high-virulence strain, and CRPA43, representing a low-virulence strain, were selected for generating survival curves (Table 4; Figure 5). In addition to exoU, three other virulence factors—exoS, exoT, and exoY—were detected across strains with varying virulence levels. Statistically significant differences were observed in the detection rates of exoU and exoS among strains with different virulence levels (P < 0.05), with high-virulence strains showing higher exoU detection rates and low-virulence strains exhibiting higher exoS detection rates (Table 5). The three virulotypes also varied across strains with different virulence levels. The exoY+/exoS-/exoT+/exoU + genotype was significantly more prevalent in high-virulence strains (P < 0.05) (Table 6).

**TABLE 4:** Survival of *Galleria mellonella* larvae at different time periods.

Group	2 h	4 h	6 h	8 h	10 h	12 h	14 h	16 h	18 h	20 h
PAO1	10	10	10	10	10	6	1	0	0	0
CRPA7	10	10	10	8	4	1	0	0	0	0
CRPA43	10	10	10	10	10	10	10	7	3	0
PBS Buffer	10	10	10	10	10	10	10	10	10	10
Blank Control	10	10	10	10	10	10	10	10	10	10

**TABLE 5:** Detection rate of T3SS virulence factors at different virulence levels (%).

Gene	Low virulence strain	Medium virulent strain	High virulence strains	*P* Value
*exoU*	0 (0/15)	12.0 (3/25)	40.0 (2/5)	**0.037**
*exoT*	100.0 (15/15)	100.0 (25/25)	100.0 (5/5)	—
*exoY*	86.7 (13/15)	88.0 (22/25)	100.0 (5/5)	1.000
*exoS*	100.0 (15/15)	88.0 (22/25)	60.0 (3/5)	**0.037**

The bolded numbers in the tables represent P values that are less than or equal to 0.05.

**TABLE 6:** Detection rate of three virulotypes at different virulence levels (%).

Genotype	Low virulence strain	Medium virulent strain	High virulence strain	*P* Value
*exoY* ^*+*^ */exoS* ^*-*^ */exoT* ^*+*^ */exoU* ^*+*^	0 (0/15)	12.0 (3/25)	40.0 (2/5)	**0.037**
*exoY* ^*-*^ */exoS* ^*+*^ */exoT* ^*+*^ */exoU* ^*-*^	13.3 (2/15)	12.0 (3/25)	0 (0/5)	1.000
*exoY* ^*+*^ */exoS* ^*+*^ */exoT* ^*+*^ */exoU* ^*-*^	86.7 (13/15)	76.0 (19/25)	60.0 (3/5)	0.401

The bolded numbers in the tables represent P values that are less than or equal to 0.05.

### 3.5 Relationship between virulence levels and drug resistance

The resistance profiles of the CRPA strains varied according to their virulence levels. Statistical analysis revealed significant differences in resistance rates to piperacillin/tazobactam, levofloxacin, and amikacin among the three virulence groups (P < 0.05). Low-virulence strains exhibited higher resistance to piperacillin/tazobactam and levofloxacin, whereas high-virulence strains demonstrated higher resistance to amikacin. Excluding polymyxin B, high-virulence strains showed lower resistance rates to the other 12 antimicrobial agents than the medium- and low-virulence strains (Table 7).

**TABLE 7:** Relationship between virulence level and drug resistance of strains (%).

Drug category	Low virulence strain	Medium virulence strain	High virulence strain	*P* Value
Piperacillin/tazobactam	86.7 (13/15)	86.7 (13/25)	40.0 (2/5)	**0.039**
Ceftazidime	66.7 (10/15)	93.3 (14/25)	40.0 (2/5)	0.632
Cefepime	53.3 (8/15)	60.0 (15/25)	40.0 (2/5)	0.757
Aztreonam	80.0 (12/15)	64.0 (16/25)	60.0 (3/5)	0.572
Imipenem	100 (15/15)	100 (25/25)	100 (5/5)	—
Meropenem	80.0 (12/15)	80.0 (20/25)	60.0 (3/5)	0.681
Ciprofloxacin	40.0 (6/15)	56.0 (14/25)	0 (0/5)	0.077
Levofloxacin	73.3 (11/15)	68.0 (17/25)	0 (0/5)	**0.010**
Amikacin	33.3 (5/15)	40.0 (10/25)	0 (0/5)	**0.023**
Ceftazidime/avibactam	40.0 (6/15)	52.0 (13/25)	40.0 (2/5)	0.759
Imipenem/relebactam	40.0 (6/15)	32.0 (8/25)	20.0 (1/5)	0.736
Cefttolozane/tazobactam	26.7 (4/15)	40.0 (6/15)	20.0 (1/5)	0.588
Polymyxin B	6.7 (1/15)	8.0 (2/25)	40.0 (2/5)	0.123

The bolded numbers in the tables represent P values that are less than or equal to 0.05.

### 3.6 Expression level analysis of virulent genes

**FIGURE 6:**
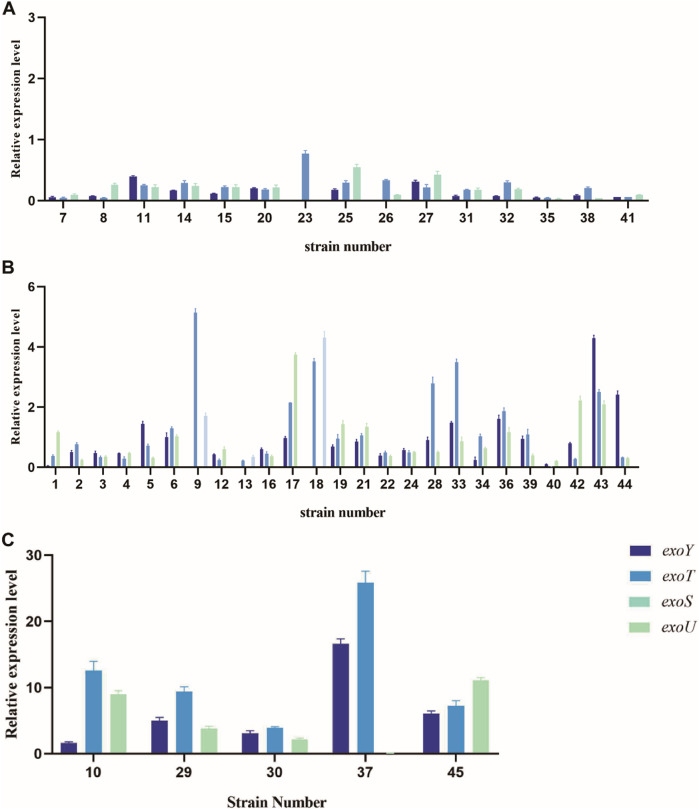
Relative expression levels of virulence genes exoY, exoT, exoU, and exoS in strains with different virulence levels. **(A)** Relative expression levels of low-virulence strains. **(B)** Relative expression levels of medium-virulence strains. **(C)** Relative expression levels of high-virulence strains.

The relative expression levels of the four virulence genes varied among the strains with different virulence levels. All experimental strains expressed exoY, exoS, exoT, and exoU; their relative expression levels are presented in Figure 6. Some strains carried virulence genes, but did not express them. Notably, high-virulence strains exhibited significantly higher expression levels of these virulence genes than strains with medium or low virulence.

## 4 Discussion

*Pseudomonas aeruginosa* is a common nosocomial opportunistic pathogen known for its high resistance rates to commonly used clinical antimicrobial agents. As the detection of CRPA strains increases, it further complicates treatment, increasing both therapeutic challenges and patient treatment costs (Reyes et al., 2023). Moreover, the emergence of highly virulent CRPA strains significantly affects patient prognosis (Huang et al., 2018). In this study, CRPA exhibited a 100% resistance rate to imipenem, the highest among the tested antibiotics, whereas polymyxin B showed the lowest resistance rate at 6.7%, proving effective against most strains, consistent with previous research findings (Lu et al., 2021). Resistance rates to amikacin, imipenem/relabatan, and cefoperazone/tazobactam were lower at 33.3%, though all were still higher than those reported by the China Antimicrobial Surveillance Network (CHINET) in 2023.

Bacterial secretion systems are crucial for the infection and pathogenesis of CRPA, with T3SS virulence genes playing a central role in inducing acute infections and facilitating bacterial spread. These genes are also linked to bacterial colonization, survival, and replication (Gil-Gil et al., 2023). The detection rate of exoT in this study was 100%, indicating that it is a common virulence factor (Feltman et al., 2001). This suggests that exoT is essential for the T3SS to function or assist other virulence factors in exerting their pathogenic effects. Although most CRPA strains carry genes encoding T3SS, not all strains harbor all four virulence genes: exoY, exoT, exoU, and exoS (Hauser, 2009). In this study, exoT, exoY, and exoS had high detection rates, in line with findings from Sarges E et al. (Sarges et al., 2020). Additionally, the coexistence of exoS and exoU was not observed, which aligns with previous reports (Hauser, 2009). This is likely because exoU and exoS occupy the same chromosomal location, leading to the exclusion and prevention of simultaneous detection in the same strain (Park et al., 2017). Furthermore, a statistically significant difference was observed in the detection rates of exoU and exoS among the strains with different virulence levels (P < 0.05). exoU was not detected in low-virulence strains, whereas exoS was detected more frequently in these strains. This suggests that the level of virulence of CRPA strains is closely related to the specific virulence genes they carry.

The *G. mellonella* larval infection model is widely used to evaluate bacterial virulence because of its advantages, including ease of handling, low cost, a natural immune system similar to that of mammals, and lack of ethical concerns (Ten et al., 2023). In this study, experimental strains were classified into three virulence levels based on the *G. mellonella* virulence test. Virulence of bacterial strains can influence antibiotic resistance (Gervasoni et al., 2023). Significant differences in resistance rates to piperacillin/tazobactam, levofloxacin, and amikacin were observed among the strains with different virulence levels (P < 0.05), indicating that virulence levels may affect antibiotic resistance. Additionally, based on the detection of these four virulence genes, the strains were categorized into three distinct virulence genotypes. The predominant genotype, exoY^+^/exoS^+^/exoT^+^/exoU^−^, accounted for 77.8% (35/45) of the total genotypes, consistent with the findings of Kiyaga S et al. (Kiyaga et al., 2022). Previous research has indicated a potential link between virulence gene carriage and antibiotic resistance (Coskun and Dagcioglu, 1992). In this study, significant differences in the resistance rates to amitraz, meropenem, and levofloxacin were noted among strains with different virulence genotypes (P < 0.05). However, the differences in resistance to other antimicrobial agents were not statistically significant, suggesting that both the type and number of virulence genes may be associated with antibiotic resistance. Furthermore, the same virulence genotype was detected in strains with varying virulence levels, suggesting that the factors influencing the virulence of CRPA are multifaceted. These factors may include not only the types and numbers of virulence genes carried by the strains but also their expression levels (Oliveira et al., 2021). This study found that highly virulent strains had significantly higher relative expression levels of the four virulence genes. Some strains carrying certain virulence genes did not express these genes, leading to differing virulence levels. This finding suggests a correlation between virulence gene expression and virulence in these strains.

A variety of β-lactamases were detected in this study, with blaOXA-101 detected at a notably higher rate than previously reported, with detection frequencies typically ranging from 5% to 9% (Ha Rui et al., 2019; Elmansoury et al., 2025). Strains harboring blaOXA-10 family genes are often phenotypically undetectable using conventional extended-spectrum β-lactamase (ESBL) tests, potentially leading to an underestimation of the prevalence of these enzymes in routine diagnostics (Elmansoury et al., 2025). BlaOXA-101 is a member of the blaOXA-10 family, a class D β-lactamase that exhibits hydrolytic activity against penicillins, cephalosporins (Farshadzadeh et al., 2014), and carbapenems. It is associated with resistance in non-fermenting Gram-negative bacilli (Alonso-Garcia et al., 2023) and is prone to mutations, leading to the emergence of new drug-resistant phenotypes (Lasarte-Monterrubio et al., 2022; Gomis-Font et al., 2022). In this study, the strains carrying blaOXA-101 exhibited resistance to ceftazidime/avibactam, imipenem/relabactam, and cefoperazone/tazobactam, with only one strain remaining susceptible to all three enzyme inhibitors. This indicates that these drugs can no longer inhibit the enzyme produced by blaOXA-101, highlighting the need for continuous monitoring. Additionally, rare β-lactamase genes such as blaPER-1, a class A β-lactamase associated with resistance to penicillins and cephalosporins, have also been detected (Alikhani et al., 2014). Regional dissemination of blaPER-1 has been documented in several contexts. For instance, Saadi et al. identified blaPER-1 in 38.3% of *P. aeruginosa* isolates from burn patients in Algeria, particularly ESBL-producing strains (Tchakal-Mesbahi et al., 2021). A similar hospital-based study in Turkey reported a blaPER-1 detection rate of 23.3% among *P. aeruginosa* isolates (Eraç et al., 2013). It has been noted that strains carrying the blaPER-1 gene typically exhibit multidrug resistance (Papa-Ezdra et al., 2023), consistent with the findings of this study, highlighting the importance of monitoring strains carrying these resistance genes in hospital settings. Notably, the reported prevalence of blaOXA-101 and blaPER-1 varies significantly across studies. These discrepancies may result from differences in specimen types (clinical vs. environmental), regional antibiotic usage patterns, and distribution of high-risk clonal lineages capable of horizontal gene transfer. These findings emphasize the need for region-specific surveillance strategies that consider both the clinical and environmental reservoirs of resistance genes.

The current international high-risk *P. aeruginosa* clonotypes include ST235, ST111, ST244, ST357, ST308, ST175, ST277, ST654, and ST298, with ST235, ST111, and ST244 being the most prevalent in our country (Hu et al., 2021; Zhao et al., 2023). High-risk clonotypes are often associated with poor clinical outcomes, primarily because of multidrug resistance (Horcajada et al., 2019; Bai et al., 2024). Notably, ST244, recognized as one of the internationally recognized high-risk clones (such as ST235 and ST111), exhibited a high prevalence rate of 31.1% in this study, reflecting trends observed across Asia. Hu et al. (Hu et al., 2021) observed that ST244 has significant transmission potential in hospital environments across East Asia and is frequently linked to multidrug-resistant phenotypes and nosocomial infection outbreaks. Similarly, Zhao et al. (Zhao et al., 2023) identified ST244 as one of the predominant clonal types in Guangdong Province, China and was closely associated with resistance genes such as blaOXA-101. Furthermore, this study identified both ST244 and ST235 strains carrying blaOXA-101, with ST244 harboring blaPER-1, indicating that these resistance genes have already spread regionally. This underscores the need for stricter disinfection and isolation measures in departments where high-risk clones are detected as well as continuous monitoring of bacterial population dynamics to control the spread of CRPA-resistant strains.

This study has several limitations. First, it was conducted at a single center, which may limit the applicability of the findings to other geographic regions or healthcare settings. Second, the relatively small sample size of the 45 CRPA isolates may not capture the full genetic and phenotypic diversity of CRPA in a broader population. Third, the absence of longitudinal follow-up restricted the ability to assess temporal trends in resistance gene dissemination and virulence expression. Although associations between virulence genotypes, gene expression, and antimicrobial resistance were observed, causal relationships could not be definitively established. Future multicenter studies with larger sample sizes and longitudinal surveillance are required to validate and expand upon these findings.

## 5 Conclusion

Most CRPA strains are multidrug-resistant, with polymyxin B being effective against most strains. Its rational use should be prioritized to preserve its antimicrobial efficacy. The carriage and expression of T3SS virulence genes are associated with both the virulence levels of the strains and their antibiotic resistance profiles. This finding could provide novel insights into antibiotic resistance, promote the rational use of antimicrobial drugs, and optimize treatment regimens for CRPA infections. The spread of ST244, carrying the enzymes blaOXA-101 and blaPER-1, within this region highlights the importance of continuous surveillance to prevent further dissemination of this high-risk clone.

## Data Availability

The datasets used and/or analyzed during the current study can be found in the OMIX databases, accession number OMIX009958.
